# Design and Evaluation of Novel Antimicrobial and Anticancer Agents Among Tetrazolo[1,5-*c*]quinazoline-5-thione S-Derivatives

**DOI:** 10.3797/scipharm.1208-13

**Published:** 2012-10-09

**Authors:** Lyudmyla M. Antypenko, Sergey I. Kovalenko, Olexii M. Antypenko, Andrey M. Katsev, Olena M. Achkasova

**Affiliations:** 1 Department of Pharmaceutical Chemistry, Zaporizhzhya State Medical University, Mayakovsky 26 ave., 69035, Zaporizhzhya, Ukraine.; 2 Department of Pharmacy, Crimea State Medical University, Lenina 5/7 ave., 95006, Simferopol, Ukraine.; 3 Bacterial Laboratory, Zaporizhzhya Regional Hospital, Orehovskoe 10 shosse, 69600, Zaporizhzhya, Ukraine.

**Keywords:** Antifungal, Antibacterial, Bioluminescence, Anticancer, 5-R-tetrazolo[1,5-*c*]quinazoline-5-thiones

## Abstract

The novel heterocyclization of 5-(2-aminophenyl)-1*H*-tetrazole with potassium ethylxanthogenate or carbon disulfide was proposed. The potassium salt of the tetrazolo[1,5-*c*]quinazoline-5-thione was subsequently modified by alkylation with proper halogen derivatives to (tetrazolo[1,5-*c*]quinazolin-5-ylthio)alkyls, *N,N*-dialkylethylamines, 1-aryl-2-ethanones, 1-(alkyl)aryl-2-ethanols, carboxylic acids, and esters. The structures of all newly synthesized compounds were confirmed by FT-IR, UV-*vis*, LC-MS, ^1^H, ^13^C NMR, and elemental analysis data. The substances were screened for antibacterial and antifungal activities (100 μg) against *Escherichia coli, Staphylococcus aureus, Enterobacter aerogenes, Entrococcus faecalis, Pseudomonas aeruginosa, Klebsiella pneumoniae,* and *Candida albicans*. Preliminary bioluminescence inhibition tests against *Photobacterium leiognathi* Sh1 showed that substances **5.2**–**5.4**, **6.1**, **7.1** with ethanone or carboxylic acid substituents showed toxicity against bacteria cells. The substances chosen by the US National Cancer Institute (NCI) were screened for their ability to inhibit 60 different human tumor cell lines, where 2-(tetrazolo[1,5-*c*]quinazolin-5-ylthio)-1-(4-tolyl)ethanone (**5.2**), 3-(tetrazolo[1,5-*c*]quinazolin-5-ylthio)propanoic and related 3-metyl-butanoic acids (**6.2**, **6.3**), and ethyl tetrazolo[1,5-*c*]quinazolin-5-ylthio)acetate (**7.2**) showed lethal antitumor activity (1.0 μM) against the acute lymphoblastic leukemia cell line (CCRF-CEM), and substances **5.2** and **6.3** exhibited moderate anticancer properties inhibiting growth of the leukemia MOLT-4 and HL06-(TB) cell lines. The moderate antitumor activity was demonstrated in 1-(2,5-dimethoxyphenyl)-2-(tetrazolo[1,5-*c*]quinazolin-5-ylthio)ethanone (**5.4**) against the CNS cancer cell line SNB-75. Comparing the docking mode of the Gefitinib and synthesised substances on the ATP binding site of EGFR, it could be assumed that these compounds might act in the same way. The results of the investigation could be considered as a useful base for future development of potent antimicrobials and antitumor agents among tetrazolo[1,5-*c*]quinazoline-5-thione S-derivatives.

## Introduction

Investigations for new effective and less toxic antimicrobials and anticancer agents are always up-to-date. The tetrazole derivatives are quite interesting objects for synthesis as well as for pharmacological screening. Despite the fact that they weren’t found in the nature and were synthetically obtained, there are a lot of biologically active substances among their fused analogues [1–7]. We are concerned with tetrazolo[1,5-*c*]quinazoline derivatives, because only few substances with very insufficient evaluation of their biological properties are reported in literature (Scheme 1) [8–16]. It is known that the main way of the above-mentioned skeleton (**1.1**) formation is the cyclisation of 4-hydrazino-quinazolines (**1.2**) with nitritic acid or the reaction of 4-halogenoquinazolines (**1.3**) with sodium azide (Scheme 1). Also, 5-(2′-aminophenyl)-*1H*-tetrazoles **1.4** are cyclized with acetic anhydride to result in proper tetrazolo[1,5-*c*]quinazolines (**1.1**).

**Fig. 1:**
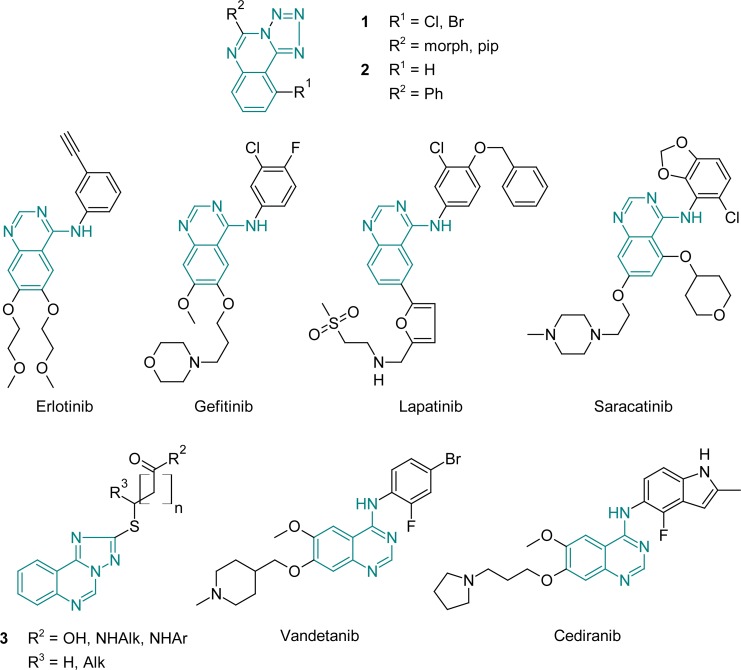
Anticancer and antimicrobial quinazoline, tetrazolo[1,5-*c*]- and triazolo[1,5-*c*]-quinazoline derivatives

Tetrazoloquinazolines are very attractive from the pharmacological point of view, because they are found to possess fungicide, pesticide, anti-allergic, bactericide, bronchodilator, antiulcer, anti-inflammatory, analgesic, and antihypertensive properties [9]. Thus, investigation of the 9-bromo/chloro-5-(morpholin-4-yl/piperidin-4-yl)-tetrazolo[1,5-*c*]quinazoline’s (**1**) antimicrobial activity against *Esherichia coli, E. faecalis, Pseudomonas aeruginosa,* and *Staphylococcus aureus* revealed that the sensitivity of Gram positive bacteria to the compounds was higher than that of Gram negative bacteria (Fig. 1) [11]. 5-Phenyltetrazolo[1,5-*c*]quinazoline (**2**) was effective against *Staphylococcus aureus* and *Esherichia faecalis* (MIC = 250 mg/L). Also, 9-bromo-5-(morpholin-4-yl)tetrazolo[1,5-*c*]-quinazoline (**1**) demonstrated antitumor activity against leukemia L1210 and column cancer Caco-2 cell lines [12]. Besides, quinazoline derivatives are important and broadly explored the structural class of EGFR inhibitors, namely the first ones found: Erlotinib (OSI-774; CP-358, Tarceva®) and Ge tinib (ZD1839; Iressa®, (Fig. 1) [17]. Also well-known anticancer drugs are Cediranib (AZD2171, Recentin®), Lapatinib (GW-0572016; Tykerb®), Vandetanib (ZD6474; Zactima®), and Saracatinib (AZD0530) (Fig. 1) [18–25].

Introduction of the sulfur atom in the 5 position of the tetrazolo[1,5-*c*]quinazoline nucleus could afford the broadening of the biological activity range. Moreover, our previous investigations in the series of similar condensed heterocycles, namely, 2-thio-[1,2,4]triazolo[1,5-*c*]quinazoline derivatives, showed that ([1,2,4]triazolo[1,5-*c*]quinazolin-2-ylthio)carboxylic acids and amides (**3**) possessed antifungal activity against *Candida albicans* and *Aspergillus niger*[26, 27]. So, the aim of this work is the purposeful formation of the tetrazolo[1,5-*c*]quinazoline-5-thione skeleton, synthesis of its S-derivatives, and screening of their antibacterial, antifungal, and antitumor properties with subsequent docking studies of the most active ones.

## Results and Discussion

### Chemistry

The tetrazolo[1,5-*c*]quinazoline (**b**) was obtained by the treatment of 4-hydrazino-quinazoline (**a**) with sodium nitrite in acetic acid at 0°C with subsequent nucleophilic degradation of its pyrimidine ring in the water solution of hydrochloric acid (1:1) to 5-(2′-aminophenyl)-1*H*-tetrazole (**c**). The latter one (**c**) was cyclized with potassium ethylxanthoganate in 2-propanol or with carbon disulfide in equimolar potassium ethoxide to produce the tetrazolo[1,5-*c*]quinazoline potassium salt **1** (Scheme 2).

Alkylation of the starting substance **1** with the methyl-/phenethyl-halogenides resulted in corresponding substances **2.1** and **2.2** at room temperature after 30 minutes. 5-[(3-Chloropropyl)thio]tetrazolo[1,5-*c*]quinazoline (**2.3**) was synthesized by the refluxing of compound **1** with unsymmetrical 1-bromo-3-chloropropane in 2-propanol. And 5-[(dialkyl-/hetarylamino)ethylthio]tetrazolo[1,5-*c*]quinazolines (**3.1**–**3.5**) were obtained by the treatment of salt **1** with appropriate 2-chloro-ethylamino(hetaryl)dialkyls hydrochlorides in equimolar potassium ethoxide (Scheme 2).

The corresponding 2-(tetrazolo[1,5-*c*]quinazolin-5-ylthio)ethanones (**5.1-5.4**), alcohols (**4.1, 4.2**), and carboxylic acid esters (**7.1**-**7.8**) were synthesized by the alkylation of starting compound **1** with appropriate halogen derivatives by refluxing in 2-propanol. Carboxylic acids (**6.1**, **6.2**) were obtained by the treatment of **1** with proper derivatives in the equimolar alkaline aqueous-2-propanol (1:1) mixture with subsequent neutralization to pH 6.5 with a dilute HCl solution (Scheme 3).

The structure of all synthesized compounds was evaluated by elemental analysis and their spectral data (FT-IR, UV, ^1^H, ^13^C-NMR, LC-MS spectra).

LC-MS data confirmed the purity of all obtained substances, demonstrating their appropriately protonated molecular ions [M + H]^+^.

The UV spectra of compounds **2.1**, **2.2**, **3.1**–**3.5**, **4.1**, **4.2**, **5.1**, **5.2**, **7.1–7.8** in methanol were characterized by absorption maximums due to the Π→ Π* transition of the benzene ring in quinazoline (high-intensive short peaks at 213–227 nm and medium-intensity peaks at 247–250 nm) and the n→Π* transition of the condensed system of tetrazole and quinazoline (two low-intensity long wavelengths at 291–339 nm).

The stretching vibrations of C-N bond of the alkylaminoalkyls (**3.1–3.5**) appeared at 1400–1007 cm^−1^; ν_s_ and δ of N-H bond − at 3068–3045 cm^−1^ and 1620–1618 cm^−1^ accordingly. FT-IR spectra of 2-(tetrazolo[1,5-*c*]quinazolin-5-ylthio)ethanones (**5.1–5.3**) were characterized by a high intensity stretching vibration of the carboxylic group at 1695-1665 cm^−1^. The intensive peak of the OH group of alcohols **4.1** and **4.2** was observed at 3271–3077 cm^−1^. In the case of intermolecular bond absorption, the signal was found at 3077–3031 cm^−1^ as the wide peak. A strong absorption of ν_C-O_ for **4.1** and **4.2** was registered at 1162–1055 cm^−1^. The signals of in-plane deformational stretchings of the O-H bond appeared at 1477–1364 cm^−1^ and out-of-plane – at 779-656 cm^−1^ for the latter compounds. Characteristic ν_C=O_ for acids **6.1**, **6.2** was fixed at 1737–1704 cm^−1^ and at 1735–1722 cm^−1^ for esters **7.1–7.8**. The spectra of **6.1–6.3** had the wide peak of the O-H group that corresponded to the stretching vibration at 3567-2519 cm^−1^ and the partially covered absorption of C-H. The stretching ν_C-O_ was detected at 1326–1202 cm^−1^ with medium intensity for acids **6.1–6.3** and as a doublet of peaks (one of them was more intensive and wider) at 1300–1015 cm^−1^ for esters **7.1–7.8**. The characteristic ν_s_ and δ of the CH_2_ – group of all synthesized substances were detected in the range of 2945–2848 cm^−1^ and 1472–1488 cm^−1^. The condensed aromatic ring was characterized by the next registered peaks: ν_CH_ (3108–3016 cm^−1^), δ_CH_ (902–649 cm^−1^), ν_CS_ (713–604 cm^−1^), and ν_CS_ and ν_CN_ (1593–710 cm^−1^).

It is known that tetrazoloquinazolines are capable of tetrazole–azide–azomethine tautomeric transformations due to the electrono-donative influence of the condensed quinazoline ring to the tetrazole one [15]. FT-IR spectral analysis of the synthesized substances (**3.1–3.5**) in solid form and in chloroform solution showed the absence of the azide group vibration at 2100 cm^−1^, which indicates the prevalence of the tetrazole form of the molecule. The low intensity vibrations of the azide tautomer at 2145 cm^−1^ and its overtone at 2198 cm^−1^ were detected when trifluoroacetic acid was added to the substances (**3.1–3.5**) in chloroform solution.

In the ^1^H NMR-spectra, the tetrazolo[1,5-*c*]quinazoline system of all synthesized substances was characterized according to the proposed structure and available literature data: the one-proton doublet of H-10 at 8.34–8.55 ppm, H-7 and H-9 as the two-proton multiplet at 8.06–7.74, and H-8 as the one-proton triplet at 7.60–7.97 ppm were observed [8]. The signal of SCH_3_ was seen in a strong field of spectra at 2.86 ppm as the three-proton singlet for substance **2.1**. The proton of the OH group was shown as a wide one-proton singlet at 14.39 ppm for alcohol **4.1** and at 13.54–12.35 ppm for acids **6.1-6.2**, and shifted to a stronger field to 11.68 ppm for **4.2**. The signal of the SCH_2_-group appeared in a strong field of the spectra as the two-proton triplet at 3.72 ppm for **2.2**, at 3.62 ppm for **2.3**, and shifted to a low field as the two-proton singlet at 5.22–5.06 ppm for alcohols **4.1, 4.2,** and to 4.72-3.40 ppm for ethanones **5.1–5.4**, and was also observed at 4.85–4.26 ppm for acid **6.1** and acid esters **7.1, 7.3, 7.4, 7.8.** The SCH-group was observed as the one-proton triplet at 5.17–5.03 ppm for **7.5**–**7.7** and as the one-proton singlet at 4.80 ppm for **7.2**. The signals of aliphatic substituents of the synthesized substances were registered in a strong field (2.89–0.86 ppm) and aromatic substituent signals were observed in a low field (7.98–7.22 ppm).

In ^13^C NMR spectra of substances **7.2** and **7.6,** the characteristic signal of the carboxylic group was registered at 170.55 ppm and 170.18 ppm respectively, and the carbonyl group of compound **5.4** at 191.50 ppm. The quinazoline C-5 was detected at 149.32–149.07 ppm, and the peak of the SCH_2_-group was registered at 38.80 ppm for **5.3**, and at 49.87–47.84 ppm for **7.2** and **7.6**, which definitely confirmed the tetrazolo[1,5-*c*]quinazoline-5-thione formation and S-regioselectivity of alkylation.

### Pharmacology

#### Photobacterium leiognathi bioluminescence inhibition

The pharmacological investigations started from the estimation of cytotoxicity of newly synthesized ([1,2,4]triazolo[1,5-*c*]quinazolin-2-ylthio)carboxylic acids by measuring the inhibition of the BL of *Photobacterium leiognathi* in acute (inhibiting BL, Table 1) and chronic action tests (inhibiting BL and growth, Table 2).

The acute action test revealed that substance **2.2** was practically toxic to bacteria at all concentrations. Substances **2.1**, **2.3**, **3.1**, **3.3**, **7.2, 7.4**–**7.7** had a moderately negative effect on bacteria BL, but compounds **3.2**, **3.4**, **4.2**, **5.3**, **7.1**, **7.8** inhibited BL to 2.4% with the increase of their concentrations. The strongest negative influence was demonstrated by 5-(2-(piperidin-1-yl)ethylthio)tetrazolo[1,5-*c*]quinazoline (**3.5**), 1-phenyl-substituted ethanons (**5.1–5.4**), and 2-(tetrazolo[1,5-*c*]quinazolin-5-ylthio)acetic acid (**6.1**).

According to the chronic action test, introduction of the methyl (**2.1**), chloroethyl (**2.3**), ethylacetate (**7.1**), methyl 2-(2,4-dichlorophenyl)acetate (**7.6**), and methyl 4-methyl-benzoate (**7.8**) groups led to the stimulation of growth and bioluminescence of bacteria (Table 2). There was practically no influence on the BL for the 5-(2-(pyrrolidin-1-yl)-ethylthio)tetrazolo[1,5-*c*]quinazoline (**3.4**) and ethyl 2-(tetrazolo[1,5-*c*]quinazolin-5-ylthio)-3-(*m*-tolyl)propanoate (**7.7**).

It is worth mentioning that substances **2.1**, **3.2**, **3.3**, **3.5**, **5.4**, and **7.3** showed the effect of the hormesis phenomenon (stimulation of activity with a lower concentration) for 0.025 and 0.1 mg/mL doses. Compounds **2.2**, **3.1**, **7.4**, **7.5** possessed moderate inhibition. The highest toxicity (inhibition Bl and growth to 0%) was shown by 4-nitro-2-((tetrazolo[1,5-*c*]quinazolin-5-ylthio)methyl)phenol (**4.2**), 1-phenyl- (**5.1**), 1-(4-methylphenyl) (**5.2**), 1-(4-ethoxyphenyl)- (**5.3**), and 1-(2,5-dimethoxyphenyl)-2-(tetrazolo[1,5-*c*]quinazolin-5-ylthio)-ethanone (**5.4**).

Thus, the SAR study revealed that the introduction of the aliphatic acid ester group resulted in the initiation of luminescent bacteria growth in the chronic test. But replacement of the alkyl group with carboxylic acid or the aromatic acid ester, 2-methyl-4-nitrophenol and ethanone substituents in the 5^th^ position of the 5-thio-tetrazolo[1,5-*c*]quinazoline led to increased cytotoxic activity against *Photobacterium leiognathi* Sh1. The high levels of growth inhibition served as a cytotoxicity marker of potential possession of the antitumor activity.

#### Antimicrobial and antifungal activities

All of the newly synthesized compounds were evaluated for their *in vitro* antibacterial activity against Gram positive bacteria (*Staphylococcus aureus*, *Enterococcus faecalis*), Gram negative bacteria (*Enterobacter aerogenes, Pseudomonas aeruginosa*, *Escherichia coli*, *Klebsiella pneumoniae*), and antifungal properties against *Candida albicans*. The agar-diffusion method was used for the determination of preliminary activity compared to well-known reference antimicrobials. All of the compounds were dissolved in DMSO in a concentration of 100 μg/disk, using inhibition zone diameter (IZD, mm) as a measure for the antimicrobial activity (Table 3).

The SAR determined that the introduction of the chloropropyl substituent in substance **2.3** was essential for its antifungal activity against *Candida albicans* like Nystatin action in the same concentration, but wasn’t enough to possess antibacterial properties. Replacing the alkyl substituent by ethylaminodialkyl for substances **3.1**–**3.3** broadened the range of their antimicrobial activity. Also, shortening of the dialkylamino fragment of substances **3.1**–**3.3** led to a moderate decrease in antimicrobial activity against *Enterococcus faecalis*, and otherwise led to an increase in antibacterial properties against *Staphylococcus aureus* and *Escherichia coli,* and antifungal properties against *Candida albicans*.

Derivatives with ethylaminohetaryl radicals (**3.4**, **3.5**) showed no antimicrobial properties. 2-(Tetrazolo[1,5-*c*]quinazolin-5-ylthio)ethanol (**4.1**) had some antimicrobial activity, but the presence of the 4-nitrophenol fragment of substance **4.2** led to the demonstration of light inhibition of *Staphylococcus aureus* growth. Introduction of the ethanone moiety into the structure of the synthesized compounds (**5.1**, **5.2**, **5.4**) had no influence on the mentioned activity. An exception was substance **5.3** with a 4-methoxyphenyl radical at the 5 position of tetrazolo[1,5-*c*]quinazoline-5-thione, which demonstrated the best results for the antibacterial and antifungal properties among all the synthesized compounds against *Staphylococcus aureus*, *Escherichia coli,* and *Candida albicans,* and was the only one that inhibited growth of *Pseudomonas aeruginosa* and *Klebsiella pneumoniae.* The impact of the halogenocarboxylic acid and ester fragments on substances **6.1**–**6.3** and **7.1**-**7.8** led to the disappearance of all their antimicrobial activities. *Enterococcus faecalis* was non-sensitive to all of the synthesized compounds.

Thus, the most active antimicrobials were found to be *N,N*-dimethyl- (**3.1**), *N,N*-diethyl- (**3.2**), *N,N*-di(isopropyl)-2-(tetrazolo[1,5-*c*]quinazolin-5-ylthio)ethanamine (**3.3**), and 1-(4-methoxyphenyl)-2-(tetrazolo[1,5-*c*]quinazolin-5-ylthio)ethanon (**5.3**). The latter one (**5.3**) with the 5-(3-chloropropylthio)tetrazolo[1,5-*c*]quinazoline (**2.3**) showed antifungal activity against *Candida albicans* like Nystatin in the 100 μg concentration.

#### Anticancer assay for preliminary in vitro testing

Among all of the newly synthesized compounds, only substances **2.1**, **2.2**, **5.1-5.4**, **6.1**-**6.3**, **7.1** were selected by the National Cancer Institute (NCI) Developmental Therapeutic Program for the *in vitro* cell line screening to investigate their anticancer activity [28–30]. The human tumor cell lines were derived from nine different cancer types: leukemia, melanoma, lung, colon, CNS, ovarian, renal, prostate, and breast cancers. Initially, a single high dose was used (1.0 μM) in the full NCI 60-cell panel. In the screening protocol, each cell line was inoculated and preincubated for 24–48 h on a microtiter plate. Then test substances were added to the plate and the culture was incubated for further 48 h. End point determinations were made with a protein binding dye, sulforhodamine B. Results for each test agent were reported as the percent growth of the treated cells when compared to the untreated control cells (Table 4).

The antitumor activity of the compounds was measured according to a value of 100 that meant no growth inhibition. A value of 30 would mean 70% growth inhibition. A value of 0 meant no net growth over the course of the experiment. A value of −30 would mean 70% lethality. A value of −100 meant all cells were dead.

Substances **5.2** (−49.78 growth percent), **6.2** (−44.08%), **6.3** (−55.61%), and **7.1** (−32.45%) showed the best of the leukemia cell line inhibition results at the CCRF-CEM line (Table 2). Also, MOLT-4 was negatively affected by substance **6.3** (46.20%) and HL-60(TB) by the **5.2** and **6.3** (36.80% and 41.25% appropriately).

Considering non-small-cell lung, colon cancer, melanoma, and ovarian cancer, they were practically insensitive to the synthesized substances except for the demonstration of the light antitumor activity by 1-(4-methoxyphenyl)-2-(tetrazolo[1,5-*c*]quinazolin-5-ylthio)-ethanone **5.3** (75.10% – HOP-92, 79.16% – HOP-62, 69.70% – NCI-H460, 81.85% – KM12, 78.16% – MALME-3M, 76.63% – SK-OV-3, 79.56% – OVCAR-4). Moreover, substance **5.3** inhibited the growth of the CNS cancer cell line SNB-75 (55.07%) and SNB-19 (75.54%). The first-mentioned CNS cell line growth was also retarded by 5-(methylthio)-tetrazolo[1,5-*c*]quinazoline **2.1** (63.81%) and 1-phenyl-2-(tetrazolo[1,5-*c*]quinazolin-5-ylthio)ethanone **5.1** (70.77%). The growth of the UO-31 renal cancer cell line was lightly delayed by almost all investigated substances (**2.1** – 61.31%, **2.2** – 82.27%, **6.1** – 70.02%, **6.2** – 75.96%, **5.1** – 63.82%, **5.3** – 81.56%, **5.4** – 78.32%, **7.1** – 85.29%). Only compounds **2.2** and **5.3** slightly affected affected the growth of the prostate and breast cancer negatively (**2.2**: PC-3 – 84.16% and **5.3**: PC-3 – 87.85%, MDA-MB-468 – 73.60%, MCF7 – 73.73%).

So, according to the SAR, introduction of the (4-tolyl)ethanone (**5.2**), acid (**6.2**, **6.3**), and ester (**7.1**) fragments into the molecule of synthesized compounds imparted them the lethal activity against acute lymphoblastic leukemia CCRF-CEM cell line, among which ethyl 2-(tetrazolo[1,5-*c*]quinazolin-5-ylthio)acetate (**7.1**) was the most active. The substitution of the 4-methyl group of substance **5.2** with the 2,5-dimethoxy group for **5.4** changedthe range of its antitumor activity greatly to the widest moderate one among all investigated compounds with the best results inhibiting the CNS cancer SNB-75 cell line. But, unfortunately all of these compounds haven’t satis ed the predetermined threshold inhibition criteria to progress to the 5-dose screen, which was designed to efficiently capture compounds with antiproliferate activity.

#### Docking, scoring, and visual inspection of synthesized substances into the ATP binding site of EGFR

To help us understand the anticancer activity of the observed compounds and guide further SAR studies, molecular docking of compounds **2.1**, **2.2**, **5.1–5.4**, **6.1**–**6.3,** and **7.1** into the ATP binding site of EGFR (2ITY.pdb) was performed [31]. The crystal structure of the enzyme with Gefitinib was obtained from the protein data bank [32]. The selected molecular targets cover some of the basic molecular mechanisms of antitumor effects. Also, the choice of the biological target was due to the literature data on the mechanism of action of 4-R-anilinoquinazolines – known anticancer products [17].

Research was conducted by flexible molecular docking as an approach to finding molecules with the affinity for a specific biological target using the software package OpenEye, including related utilities: Fred Receptor2.2.5, Vida4.1.1, Flipper, Babel3, Omega2.4.3, and Fred2.2.5 [33].

According to Consensus score, compound **6.2** showed the highest affinity for EGFR, but was still lower compared to other known drugs (Gefitinib, Lapatinib). The calculated values are presented in Table 5.

**Fig. 2:**
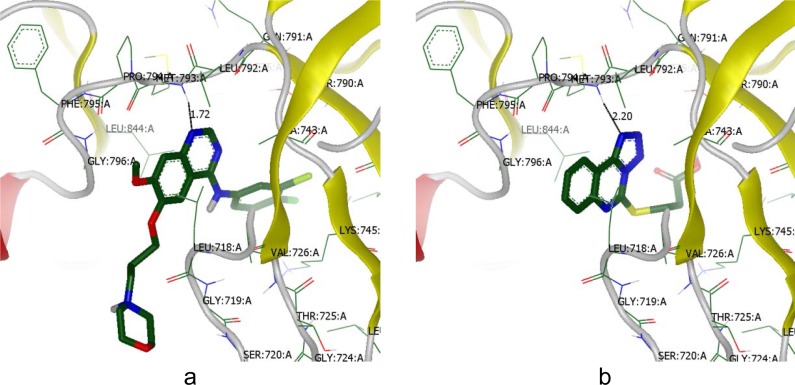
a: Interaction of Gefitinib with the binding site of EGFR; b: Interaction of compound 6.2 with the binding site of EGFR. H-bonds are shown as black lines.

Molecular docking with visual inspection indicated that compound **6.2** was nicely bound to the EGFR kinase with a hydrogen bond (marked by black color line): N···NH/Met 793 (distance 2.20 Å), while Gefitinib had the same but a shorter hydrogen bond (distance 1.72 Å) (Fig.2).

Thus, after comparing the docking mode, it could be postulated that the designed compounds might act on the same enzyme target where Gefitinib acted.

## Conclusions

The efficient and general procedure for the synthesis of the novel tetrazolo[1,5-*c*]-quinazoline-5-thione potassium salt **1** *via* cyclization of 5-(2′-aminophenyl)-*1H*-tetrazole with potassium ethylxanthogenate in 2-propanol, or with carbon disulfide in potassium ethoxide solution, has been developed. The products of the alkylation of substance **1** with proper halogenoalkyls, 2-chloro-ethylamino(hetaryl)dialkyls, halogenoethanons, halogeno-alcohols, halogenocarboxylic acids, and their esters were elucidated by FT-IR, LC-MS, ^1^H, ^13^C NMR, and elemental analysis data. The subsequent screening for antibacterial and antifungal activities in the concentration 100 μg revealed that the most active substances were *N,N*-dimethyl- (**3.1**), *N,N*-diethyl- (**3.2**), *N,N*-di(isopropyl)-2-(tetrazolo[1,5-*c*]quinazolin-5-ylthio)ethanamine (**3.3**), and 1-(4-methoxyphenyl)-2-(tetrazolo[1,5-*c*]quinazolin-5-ylthio)-ethanone (**5.3**). The SAR revealed that shortening of the dialkylamino fragment of substances **3.1**–**3.3** leads to a moderate decrease in antimicrobial activity against *Enterococcus faecalis*, and otherwise leads to an increase in activity against *Staphylococcus aureus* and *Escherichia coli,* and antifungal activity against *Candida albicans.* Introduction of the 4-methoxyphenyl group at 5 position of tetrazolo[1,5-*c*]-quinazoline-5-thione (**5.3**) resulted in the synthesis of the best antimicrobial agent among all investigated substances that even moderately inhibited the growth of *Pseudomonas aeruginosa* and *Klebsiella pneumoniae.* Preliminary BL inhibition tests against *Photobacterium leiognathi* Sh1 showed that substances (**5.2**–**5.4**, **6.1**, **7.1**) with ethanon or carboxylic acid substituents had toxicity against bacteria cells. Thus, screening of the antitumor activity showed that in the concentration 1.0 μM 2-(tetrazolo[1,5-*c*]quinazolin-5-ylthio)-1-(4-tolyl)-ethanone (**5.2**), 3-(tetrazolo[1,5-*c*]quinazolin-5-ylthio)propanoic and 3-methyl-butanoic acids (**6.2**, **6.3**), and ethyl tetrazolo[1,5-*c*]quinazolin-5-ylthio)acetate (**7.1**) exhibited lethal activity against the acute lymphoblastic leukemia CCRF-CEM cell line, and substances **5.2** and **6.3** exhibited moderate anticancer properties against the leukemia MOLT-4 and HL06-(TB) cell lines. The 1-(2,5-dimethoxyphenyl)-2-(tetrazolo-[1,5-*c*]quinazolin-5-ylthio)ethanone **5.4** demonstrated the widest range of antitumor activity, inhibiting some cancer cell lines of almost all types with the best negative influence on the CNS cancer cell line SNB-75. So, the introduction of the molecules 3-propanoic and 3–methyl-butanoic acid (**6.2**, **6.3**), ethyl acetate (**7.2**), and (4-methoxyphenyl)ethanone (**5.3**) fragments were the best choice for possessing anticancer activities. Although the results of the antimicrobial activity are not very potent, the anticancer results are quite promising. Compared to the docking mode, it could be assumed that the synthesized substances might act on the ATP binding site of EGFR like Gefitinib does. And they could be used for advanced QSAR analysis based on *in vitro* biological activity investigations for the further purposeful optimization of the leading compounds in the more effective antimicrobials because of the ever-mounting problem of microorganisms’ resistance as well as novel anticancer agents.

## Experimental

Melting points were determined in open capillary tubes in a Thiele’s apparatus and were uncorrected. The elemental analyses (C, H, N, S) were performed using the ELEMENTAR vario EL cube analyzer. UV-vis spectra (190–400 nm) were recorded on the Analytic Jena UV-vis spectrophotometer Specord 200 in methanol. The IR spectra (4000–600 cm^−1^) were recorded on a Bruker ALPHA FT-IR spectrometer using a module ATR eco ZnSe. ^1^H NMR spectra (400 MHz and 500 MHz) were recorded on the Varian-Mercury 400 and Bruker Avance DRX-500 spectrometers with SiMe_4_ as the internal standard in DMSO*-d_6_* solution. The LC-MS was recorded using a chromatography/mass spectrometric system which consisted of the high-performance liquid chromatograph “Agilent 1100 Series” equipped with the diode-matrix and mass-selective detector “Agilent LC/MSD SL” (atmospheric pressure chemical ionization – APCI). The purity of all obtained compounds was checked by ^1^H NMR and LC-MS.

Tetrazolo[1,5-*c*]quinazoline (**b**) and 5-(2-aminophenyl)-*1H*-tetrazole (**c**) were obtained by the reported in literature procedures [10].

Other starting materials and solvents were obtained from commercially available sources and used without additional purification.

### General Procedure for the Synthesis of Tetrazolo[1,5-c]quinazolin-5(1H)-thione Potassium Salt (1)

Potassium *O*-ethylxanthogenate (6.2 mmol) was added to the solution of 5-(2′-aminophenyl)-*1H*-tetrazole **c** (6.2 mmol) in 2-propanol (10 mL). The resulting mixture was refluxed for 2 h. After cooling to room temperature, the crystalline precipitate was filtered and dried.

#### Potassium salt of Tetrazolo[1,5-c]quinazoline-5(1H)-thione (**1**)

Yellow solid in 65.60% yield, mp 240–245°C. ^1^H NMR (500 MHz): δ (ppm) 8.20 (d, *J* = 7.78 Hz, 1H, H-10), 7.69 (dd, *J* = 8.22, 7.12 Hz, 1H, H-7), 7.56 (t, *J* = 8.34 Hz, 1H, H-9), 7.36 (t, *J* = 7.45 Hz, 1H, H-8). IR (cm^−1^): 1621, 1566, 1504, 1472, 1452, 1442, 1358, 1323, 1314, 1302, 1274, 1253, 1211, 1136, 1113, 1103, 1040, 1006, 985, 971, 925, 851, 767, 716, 671, 631, 607. UV (nm): 213, 234, 368. *Anal*. Calcd. for C_8_H_5_N_5_S: C, 47.28; H, 2.48; N, 34.46; S, 15.78. Found: C, 47.25; H, 2.53; N, 34.45; S, 15.75.

### General Procedure for the Synthesis of 5-(alkylthio)tetrazolo[1,5-c]quinazolines (2.1–2.3)

Proper chloroalkane(phenethyl) (4.4 mmol) was added to the suspension of potassium salt of tetrazolo[1,5-*c*]quinazolin-5(1*H*)-thione **1** (0.94 g, 4 mmol) in 15 mL of 2-propanol. The resulting mixture was refluxed for 30 minutes. After cooling, the precipitate was filtered out, washed with water, recrystallized from 2-propanol, and dried.

#### 5-(Methylthio)tetrazolo[1,5-c]quinazoline (**2.1**)

White solid in 80.55% yield, mp 158–160°C. ^1^H NMR (400 MHz): δ (ppm) 8.51 (d, *J* = 7.20 Hz, 1H, H-10), 8.12-7.91 (m, 2H, H-7,9), 7.80 (t, *J* = 6,57 Hz, 1H, H-8), 2.86 (s, 3H, CH_3_). IR (cm^−1^): 3383, 2973, 2917, 2849, 1725, 1616, 1586, 1516, 1486, 1472, 1446, 1379, 1311, 1275, 1251, 1208, 1156, 1135, 1101, 1035, 985, 964, 903, 875, 846, 761, 712, 686, 640, 630. UV (nm): 222, 248, 291, 322. *Anal*. Calcd. for C_9_H_7_N_5_S C, 49.76; H, 3.25; N, 32.24; S, 14.76. Found: C, 49.79; H, 3.23; N, 32.21; S, 14.79.

#### 5-(Phenethylthio)tetrazolo[1,5-c]quinazoline (**2.2**)

Light-brown solid in 70.65% yield, mp 118–120°C. ^1^H NMR (400 MHz): δ (ppm) 8.50 (d, *J* = 7.71 Hz, 1H, H-10), 8.06 (d, *J* = 7.85 Hz, 1H, H-7), 8.00 (t, *J* = 7.56 Hz, 1H, H-9), 7.81 (t, *J* = 6.86 Hz, 1H, H-8), 7.41 (d, *J* = 6.47 Hz, 2H, Ph-3,5), 7.36 (t, *J* = 6.59 Hz, 2H, Ph-2,6), 7.29-7.22 (m, 1H, Ph-4), 3.72 (t, *J* = 6.42 Hz, 2H, SCH_2_), 3.17 (t, *J* = 6.52 Hz, 2H, CH_2_Ph). IR (cm^−1^): 3063, 3026, 2921, 2850, 1951, 1876, 1723, 1619, 1586, 1561, 1487, 1473, 1451, 1400, 1382, 1321, 1277, 1262, 1230, 1214, 1162, 1138, 1108, 1072, 1036, 981, 967, 912,848, 828, 773, 759, 712, 695, 642. UV (nm): 222, 249, 294, 323. LC-MS: *m/z* = 308 [M + H]^+^. Anal. Calcd. for C_16_H_13_N_5_S C, 62.52; H, 4.26; N, 22.78; S, 10.43. Found: C, 62.54; H, 4.28; N, 22.74; S, 10.40.

#### 5-[(3-Chloropropyl)thio]tetrazolo[1,5-c]quinazoline (**2.3**)

Light-brown solid in 58.09% yield, mp 76–78°C. ^1^H NMR (400 MHz): δ (ppm) 8.52 (d, *J* = 7.81 Hz, 1H, H-10), 8.02 (m, 2H, H-7,9), 7.82 (t, *J* = 7.24 Hz, 1H, H-8), 3.85 (t, *J* = 6.21 Hz, 2H, CH_2_Cl), 3.62 (t, *J* = 6.84 Hz, 2H, SCH_2_), 2.33 (t, *J* = 6.30 Hz, 2H, SCH_2_*CH_2_*). IR (cm^−1^): 3096, 3066, 3041, 3016, 2945, 2913, 2848, 1615, 1585, 1557, 1488, 1472, 1445, 1378, 1351, 1322, 1310, 1263, 1210, 1162, 1136, 1100, 1076, 1033, 1022, 967, 951, 875, 861, 799, 772, 712, 695, 683, 641, 631, 604. LC-MS: *m/z* = 280 [M + H]^+^. Anal. Calcd. for C_11_H_10_ClN_5_S: C, 47.23; H, 3.60; N, 25.03; S, 11.46. Found: C, 47.20; H, 3.63; N, 25.06; S, 11.44.

### General Procedure for the Synthesis of N,N-dialkyl-2-(tetrazolo[1,5-c]quinazoline-5-ylthio)alkylamines (3.1–3.5)

To a solution of triethylamine 0.83 mL (6 mmol) in 20 mL of dioxane was added an appropriate hydrochloride chloroethyl(hetaryl)amine (6 mmol) and potassium salt of tetrazolo[1,5-*c*]quinazolin-5(1*H*)-thione **1** 2.0 g (6 mmol).The mixture was refluxed for 3 h. After cooling, the precipitate was filtered out, washed with water, recrystallized from 2-propanol, and dried.

#### N,N-Dimethyl-2-(tetrazolo[1,5-c]quinazolin-5-ylthio)ethanamine (**3.1**)

White solid in 58.39% yield, mp 100–102°C. ^1^H NMR (400 MHz): δ (ppm) 8.50 (d, *J* = 7.74 Hz, 1H, H-10), 7.99 (m, H-7,9), 7.80 (t, *J* = 7.66 Hz, 1H, H-8), 3.63 (t, *J* = 6.46 Hz, 2H, SCH_2_), 2.84-2.68 (m, 2H, CH_2_N), 2.28 (s, 6H, (CH_3_)_2_). IR (cm^−1^): 2932, 2861, 2821, 2780, 1620, 1588, 1562, 1532, 1488, 1477, 1453, 1425, 1380, 1326, 1294, 1274, 1252, 1218, 1153, 1139, 1127, 1111, 1097, 1063, 1034, 1015, 980, 962, 900, 871, 765, 711, 687, 638, 605. UV (nm): 222, 249, 292, 322. LC-MS: *m/z* = 275 [M + H]^+^. Anal. Calcd. for C_12_H_14_N_6_S: C, 52.54; H, 5.14; N, 30.63; S, 11.69. Found: C, 52.55; H, 5.10; N, 30.65; S, 11.70.

#### N,N-Diethyl-2-(tetrazolo[1,5-c]quinazolin-5-ylthio)ethanamine (**3.2**)

White solid in 59.52% yield, mp 176–178°C. ^1^H NMR: δ (ppm) 8.52 (d, *J* = 7.76 Hz, 1H, H-10), 8.00 (m, 2H, H-7,9), 7.80 (t, 1H, H-8), 3.60 (t, 2H, *J* = 6.84 Hz, SCH_2_), 2.80 (m, 2H, CH_2_N), 2.65 (m, 4H, N(CH_2_)_2_), 1.05 (m, 6H, (CH_3_)_2_). IR (cm^−1^): 2968, 2923, 2874, 2850, 2799, 2733, 2585, 2484, 1619, 1587, 1563, 1487, 1471, 1453, 1433, 1382, 1357, 1318, 1289, 1278, 1212, 1198, 1178, 1158, 1142, 1105, 1070, 1035, 1007, 977, 966, 914, 876, 789, 780, 772, 732, 714, 687, 645. UV (nm): 222, 248, 292, 321. LC-MS: *m/z* = 303 [M + H]^+^. Anal. Calcd. for C_14_H_18_N_6_S: C, 55.61; H, 6.00; N, 27.79; S, 10.60. Found: C, 55.64; H, 6.04; N, 27.75; S, 10.56.

#### N,N-Diisopropyl-2-(tetrazolo[1,5-c]quinazolin-5-ylthio)ethanamine (N-Isopropyl-N-[2-(tetrazolo[1,5-c]quinazolin-5-ylthio)ethyl]propan-2-amine, **3.3**)

White solid in 63.55% yield, mp 178–180°C. ^1^H NMR (400 MHz): δ (ppm) 8.52 (d, *J* = 7.85 Hz, 1H, H-10), 7.99 (m, 2H, H-7,9), 7.81 (t, *J* = 7.66 Hz, 1H, H-8), 3.48 (m, 2H, SCH_2_), 3.15 (m, 2H, CH_2_N), 2.89 (m, 2H, 2xN(CH)), 1.65-0.86 (m, 12H, 4xCH_3_). IR (cm^−1^): 3242, 3062, 3041, 2960, 2921, 2850, 2807, 2661, 2569, 2513, 1669, 1618, 1588, 1563, 1491, 1469, 1452, 1400, 1381, 1361, 1324, 1279, 1255, 1215, 1162, 1131, 1106, 1073, 1036, 965, 921, 823, 786, 778, 741, 713, 689, 644. UV (nm): 221, 247, 291, 321. LC-MS: *m/z* = 331 [M + H]^+^. Anal. Calcd. for C_16_H_22_N_6_S: C, 58.16; H, 6.71; N, 25.43; S, 9.70. Found: C, 58.20; H, 6.68; N, 25.44; S, 9.71.

#### 5-{[2-(Pyrrolidin-1-yl)ethyl]thio}tetrazolo[1,5-c]quinazoline (**3.4**)

White solid in 58.26% yield, mp 92–94°C. ^1^H NMR (400 MHz): δ (ppm) 8.51 (d, *J* = 7.58 Hz, 1H, H-10), 8.01 (m, 2H, H-7,9), 7.81 (t, *J* = 7.66 Hz, 1H, H-8), 3.66 (t, *J* = 6.43 Hz, 2H, SCH_2_), 2.93 (t, *J* = 6.02 Hz, 2H, CH_2_N), 2.62 (s, 4H, Pyr-2,5), 1.74 (s, 4H, Pyr-3,4). IR (cm^−1^): 2963, 2950, 2929, 2908, 2880, 2807, 2787, 2741, 1620, 1588, 1563, 1489, 1478, 1452, 1428, 1382, 1357, 1325, 1289, 1276, 1248, 1229, 1212, 1184, 1155, 1110, 1097, 1035, 978, 970, 907, 895, 878, 808, 767, 710, 687, 639, 606. UV (nm): 222, 248, 294, 322. LC-MS: *m/z* = 301 [M + H]^+^. Anal. Calcd. for C_14_H_16_N_6_S: C, 55.98; H, 5.37; N, 27.98; S, 10.67. Found: C, 55.96; H, 5.40; N, 27.95; S, 10.69.

#### 5-{[2-(Piperidin-1-yl)ethyl]thio}tetrazolo[1,5-c]quinazoline (**3.5**)

Light-brown solid in 64.41% yield, mp 80–81°C. ^1^H NMR (500 MHz): δ (ppm) 8.52 (d, *J* = 8.16 Hz, 1H, H-10), 8.01 (m, 2H, H-7,9), 7.81 (ddd, *J* = 8.04, 1H, H-8), 3.66 (t, *J* = 6.88 Hz, 2H, SCH_2_) 2.81 (s, 2H, CH_2_N), 2.51 (m, 4H, Pip-2,6) 1.64-1.46 (m, 6H, Pip-3,4,5). IR (cm^−1^): 3104, 3068, 3045, 2951, 2922, 2854, 2800, 2753, 2724, 2680, 2639, 1619, 1586, 1563, 1489, 1474, 1451, 1442, 1403, 1382, 1352, 1326, 1303, 1276, 1257, 1242, 1156, 1110, 1065, 1038, 1023, 1007, 995, 981, 971, 907, 876, 856, 768, 730, 712, 689, 644, 632. UV (nm): 222, 249, 294, 322. LC-MS: *m/z* = 315 [M + H]^+^. Anal. Calcd. for C_15_H_18_N_6_S: C, 57.30; H, 5.77; N, 26.73; S, 10.20. Found: C, 57.27; H, 5.79; N, 26.71; S, 10.24.

### General Procedure for the Synthesis of alcohols 4.1 4.2)

To a suspension of tetrazolo[1,5-*c*]quinazolin-5(1*H*)-thione, potassium salt **1** 0.95 g (4 mmol) in 10 mL of 2-propanol was added appropriate chloroalcohol (4.4 mmol) in 20 mL of propan-2-ol. The mixture was refluxed for 3 h. After cooling, the precipitate was filtered out, washed with water, recrystallized from 2-propanol, and dried.

#### 2-(Tetrazolo[1,5-c]quinazolin-5-ylthio)ethanol (**4.1**)

White solid in 70.38% yield, mp 210–212°C. ^1^H NMR (400 MHz): δ (ppm) 14.39 (br.s., 1H, OH), 8.34 (d, *J* = 7.89 Hz, 1H, H-10), 7.88 (d, *J* = 7.82 Hz, 1H, H-7), 7.71 (t, *J* = 8.28 Hz, 1H, H-9), 7.60 (t, *J* = 7.60 Hz, 1H, H-8), 3.40 (s, 4H, (CH_2_)_2_). IR (cm^−1^): 3271, 3236, 3220, 3180, 3142, 3108, 3077, 3031, 2996, 2927, 2761, 1753, 1630, 1593, 1557, 1537, 1519, 1477, 1460, 1364, 1316, 1290, 1273, 1229, 1162, 1111, 1055, 987, 966, 955, 902, 865, 779, 753, 733, 707, 690, 656, 632, 610. UV (nm): 227, 250, 310, 339. Anal. Calcd. for C_10_H_9_N_5_OS C, 48.57; H, 3.67; N, 28.32; S, 12.97. Found: C, 48.60; H, 3.65; N, 28.35; S, 13.99.

#### 4-Nitro-2-[(tetrazolo[1,5-c]quinazolin-5-ylthio)methyl]phenol (**4.2**)

White-yellowish solid in 81.13% yield, mp 204–206°C. ^1^H NMR (400 MHz): δ (ppm) 11.68 (br.s., 1H, OH), 8.51 (d, *J* = 7.82 Hz, 1H, H-10), 8.65 (d, *J* = 2.64 Hz, 1H, Ph-6), 8.16 (d, *J* = 8.15 Hz, 1H, Ph-4), 8.06 (m, 2H, H-7,9), 7.82 (t, *J* = 7.51 Hz, 1H, H-8), 7.04 (d, *J* = 8.98 Hz, 1H, Ph-3), 4.74 (s, 2H, SCH_2_). IR (cm^−1^): 3111, 2917, 2850, 1615, 1589, 1521, 1489, 1454, 1440, 1403, 1380, 1334, 1291, 1279, 1246, 1233, 1172, 1148, 1128, 1111, 1079, 1051, 1023, 972, 936, 908, 883, 843, 825, 780, 742, 713, 681, 669, 661, 639. UV (nm): 223, 247, 295, 322. LC-MS: *m/z* = 355 [M + H]^+^. Anal. Calcd. for C_15_H_10_N_6_O_3_S C, 50.84; H, 2.84; N, 23.72; S, 9.05. Found: C, 50.87; H, 2.81; N, 23.75; S, 9.08.

### General Procedure for the Synthesis of 1-Aryl-2-(tetrazolo[1,5-c]quinazolin-5-ylthio)ethanones (5.1–5.4)

To a solution of tetrazolo[1,5-*c*]quinazolin-5(1*H*)-thione potassium salt **1** 1.0 g (4 mmol) in 5 mL of water was added the solution of appropriate chloroethanones (4.4 mmol) in 5 mL of 2-propanol. The resulting mixture was heated for 3 h. After cooling, the precipitate was filtered out, washed with water, recrystallized from 2-propanol, and dried.

#### 1-Phenyl-2-(tetrazolo[1,5-c]quinazolin-5-ylthio)ethanone (**5.1**)

Light-brown solid in 50.53% yield, mp 158–160°C. ^1^H NMR (400 MHz): δ (ppm) 8.51 (d, *J* = 7.79 Hz, 1H, H-10), 8.18 (m, 2H, H-7,9), 7.92 (t, *J* = 7.67 Hz, 1H, H-8), 7.78 (qd, *J* = 7.67 Hz, 2H, Ph-2,6), 7.65 (t, *J* = 6.76 Hz, 3H, Ph-3,4,5), 5.22 (s, 2H, SCH_2_). IR (cm^−1^): 3061, 3036, 3004, 2957, 2916, 2848, 1997, 1961, 1923, 1882, 1843, 1675, 1619, 1586, 1562, 1536, 1492, 1478, 1448, 1381, 1326, 1293, 1276, 1214, 1195, 1186, 1164, 1106, 1079, 1036, 993, 981, 969, 884, 787, 770, 755, 714, 688, 649, 629. UV (nm): 224, 248, 291, 321. LC-MS: *m/z* = 322 [M + H]^+^. Anal. Calcd. for C_16_H_11_N_5_OS: C, 59.80; H, 3.45; N, 21.79; S, 9.98. Found: C, 59.84; H, 3.41; N, 21.77; S, 9.95.

#### 2-(Tetrazolo[1,5-c]quinazolin-5-ylthio)-1-(p-tolyl)ethanone (**5.2**)

Light-brown solid in 74.54% yield, mp 154–158°C. ^1^H NMR (400 MHz): δ (ppm) 8.51 (d, *J* = 7.76 Hz, 1H, H-10), 8.07 (m, H-7,9), 7.98-7.84 (m, 2H, Ph-2,6), 7.79 (t, *J* = 7.46 Hz, 1H, H-8), 7.44 (d, *J* = 7.62 Hz, 2H, Ph-3,5), 5.19 (s, 2H, SCH_2_), 2.42 (s, 3H, CH_3_). IR (cm^−1^): 3065, 2952, 2915, 2849, 1999, 1960, 1941, 1923, 1844, 1802, 1695, 1673, 1618, 1601, 1583, 1538, 1489, 1479, 1453, 1402, 1381, 1319, 1297, 1276, 1256, 1217, 1205, 1195, 1183, 1163, 1147, 1121, 1107, 1037, 996, 967, 897, 885, 848, 808, 785, 772,740, 713, 687, 668, 643, 604. UV (nm): 224, 249, 294, 322. LC-MS: *m/z* = 336 [M + H]^+^. Anal. Calcd. for C_17_H_13_N_5_OS: C, 60.88; H, 3.91; N, 20.88; S, 9.56. Found: C, 60.85; H, 3.94; N, 20.85; S, 9.54.

#### 1-(4-Methoxyphenyl)-2-(tetrazolo[1,5-c]quinazolin-5-ylthio)ethanone (**5.3**)

White solid in 71.15% yield, mp 155–157°C. ^1^H NMR (500 MHz): δ (ppm) 8.52 (d, *J* = 7.97 Hz, 1H, H-10), 8.16 (d, *J* = 8.21 Hz, 2H, H-7, Ph-2), 7.94 (t, *J* = 7.49 Hz, 1H, H-9), 7.80 (t, *J* = 7.58 Hz, 1H, H-8), 7.73 (d, *J* = 8.15 Hz, Ph-6), 7.16 (d, *J* = 8.26 Hz, 2H, Ph-3,5), 5.18 (s, 2H, SCH_2_), 3.91 (s, 3H, OCH_3_). ^13^C NMR (500 MHz): δ (ppm) 191,50 (C=O), 164,15 (Ph-4), 149,32 (C-5), 146,44 (C-7a), 143,42 (C-11), 134,32 (C-8), 131,39 (Ph-2,6), 129,11 (Ph-1), 129,02 (C-7), 127,42 (C-9), 124,78 (C-10), 114,64 (C-10a), 114,38 (Ph-3,5), 56,19 (OCH3), 38,80 (SCH). IR (cm^−1^): 3059, 3007, 2968, 2915, 2838, 1665, 1616, 1600, 1576, 1489, 1479, 1453, 1419, 1382, 1328, 1312, 1302, 1277, 1254, 1200, 1176, 1108, 1033, 1008, 994, 967, 881, 829, 800, 788, 771, 715, 686, 606. LC-MS: not soluble. Anal. Calcd. for C_18_H_15_N_5_O_3_S C, 56.68; H, 3.96; N, 18.36; S, 8.41. Found: C, 56.70; H, 3.94; N, 18.38; S, 8.44.

#### 1-(2,5-Dimethoxyphenyl)-2-(tetrazolo[1,5-c]quinazolin-5-ylthio)ethanone (**5.4**)

White solid in 77.46% yield, mp 180–186°C. ^1^H NMR (500 MHz): δ (ppm) 8.52 (d, *J* = 7.85 Hz, 1H, H-10), 7.97 (t, *J* = 7.66 Hz, 1H, H-7), 7.81 (t, *J* = 7.59 Hz, 1H, H-9), 7.78 (t, *J* = 8.13 Hz, 1H, H-8), 7.28-7.19 (m, 3H, Ph-3,4,5), 5.06 (s, 2H, SCH_2_), 4.00 (s, 3H, 2-OCH_3_), 3.76 (s, 3H, 5-OCH_3_). IR (cm^−1^): 3059, 3016, 2988, 2945, 2833, 1665, 1619, 1585, 1563, 1494, 1475, 1460, 1453, 1413, 1383, 1372, 1328, 1286,1264, 1222, 1155,1137, 1107, 1048, 1037, 1023, 980, 967, 913, 887, 869, 822, 771, 710, 689, 647, 632, 621. LC-MS: *m/z* = 382 [M + H]^+^. Anal. Calcd. for C_18_H_15_N_5_O_3_S C, 56.68; H, 3.96; N, 18.36; S, 8.41. Found: C, 56.70; H, 3.94; N, 18.38; S, 8.44.

### General Procedure for the Synthesis of (Tetrazolo[1,5-c]quinazolin-5-ylthio)-carboxylic Acids (6.1–6.3)

To the solution of tetrazolo[1,5-*c*]quinazolin-5(1*H*)-thione, potassium salt **1** 1.0 g (4 mmol) in 10 mL of H_2_O was added the solution of halogenocarboxylic acid (4.4 mmol) in 15 mL of water–2-propanol (1:1) solution with KOH (4 mmol). The mixture was stirred at room temperature for 4 h. All insoluble materials were filtered from the reaction mixture. The resulting solution was adjusted to pH 3–4 by adding 10% aqueous HCl solution. The precipitate was filtered, dried, and recrystallized from 2-propanol.

#### (Tetrazolo[1,5-c]quinazolin-5-ylthio)acetic acid (**6.1**)

White solid in yield 76.66%, mp 156–158°C. ^1^H NMR (400 MHz): δ (ppm) 13.54–12.90 (s, 1H, COOH), 8.47 (d, *J* = 7.84 Hz, 1H, H-10), 7.95 (qd, *J* = 8.14 Hz, 2H, H-7,9), 7.80 (t, J = 7.26, 1H, H-8), 4.35 (s, 2H, SCH_2_). IR (cm^−1^): 2997,2913, 2711, 2593, 1705, 1619, 1591, 1560, 1521, 1493, 1476, 1455, 1423, 1379, 1328, 1303, 1278, 1251, 1221, 1175, 1142, 1111, 1040, 982, 971, 922, 911, 785, 779, 753, 713, 690, 673, 647, 632. UV (nm): 222, 248, 290, 321. LC-MS: *m/z* = 262 [M + H]^+^. Anal. Calcd. for C_10_H_7_N_5_O_2_S: C, 45.97; H, 2.70; N, 26.81; S, 12.27. Found: C, 46.00; H, 2.73; N, 26.84; S, 12.23.

#### 3-(Tetrazolo[1,5-c]quinazolin-5-ylthio)propanoic acid (**6.2**)

White solid in yield 82.02%, mp 170–172°C. ^1^H NMR: δ (ppm) 12.35 (s, 1H, COOH), 8.52 (d, *J* = 7.85 Hz, 1H, H-10), 8.00 (m, 2H, H-7,9), 7.78 (t, *J* = 7.24 Hz, 1H, H-8), 3.65 (t, 2H, SCH_2_), 2.88 (t, 2H, CH_2_). IR (cm^−1^): 2968, 2923, 2874, 2850, 2799, 2733, 2585, 2484, 1619, 1587, 1563, 1487, 1471, 1453, 1433, 1382, 1357, 1318, 1289, 1278, 1212, 1198, 1178, 1158, 1142, 1105, 1070, 1035, 1007, 977, 966, 914, 876, 789, 780, 772, 732, 714, 687, 645. LC-MS: *m/z* = 303 [M + H]^+^. Anal. Calcd. for C_14_H_18_N_6_S: C, 55.61; H, 6.00; N, 27.79; S, 10.60. Found: C, 47.95; H, 3.34; N, 25.41; S, 11.62.

#### 3-Methyl-2-(tetrazolo[1,5-c]quinazolin-5-ylthio)butanoic acid (**6.3**)

White solid in yield 71.70%, mp 169–172°C. IR (cm^−1^): 2966, 2928, 2895, 2871, 2836, 2635, 2521, 1709, 1629, 1619, 1590, 1559, 1539, 1520, 1493, 1477, 1460, 1451, 1429, 1390, 1368, 1326, 1300, 1291, 1273, 1256, 1162, 1110, 1055, 1038, 981, 966, 923, 846, 770, 752, 723, 713, 690, 9555, 632, 609. Anal. Calcd. for C_13_H_13_N_5_OS: C, 51.47; H, 4.32; N, 23.09; S, 10.57. Found: C, 51.50; H, 4.29; N, 23.12; S, 10.56.

### General Procedure for the Synthesis of (Tetrazolo[1,5-c]quinazoline-5-ylthio)-carboxylic Acid Esters (7.1–7.8)

The appropriate halogenocarboxylic acid ester (4.3 mmol) was added to a suspension of tetrazolo[1,5-*c*]quinazolin-5(1*H*)-thione potassium salt **1** 1.05 g (4.3 mmol) in 10 mL of 2-propanol. The resulting mixture was heated for 4 h. After cooling to room temperature, a five-fold excess of H_2_O was added. The crystalline precipitate was filtered, dried, and recrystallized from 2-propanol.

#### Methyl (tetrazolo[1,5-c]quinazolin-5-ylthio)acetate (**7.1**)

White solid in 52.67% yield, mp 109–111°C. ^1^H NMR (400 MHz): δ (ppm) 8.53 (d, *J* = 7.78 Hz, 1H, H-10), 8.07-7.92 (m, 2H, H-7,9), 7.84 (t, *J* = 7.32 Hz, 1H, H-8), 4.44 (s, 2H, SCH_2_), 3.76 (s, 3H, OCH_3_). IR (cm^−1^): 3069, 3031, 2990, 2917, 2848, 2000, 1969, 1879, 1734, 1616, 1588, 1516, 1489, 1477, 1451, 1433, 1386, 1372, 1325, 1299, 1277, 1248, 1201, 1167, 1106, 1035, 1022, 992, 980, 966, 966, 888, 778, 712, 685, 644. UV (nm): 221, 248, 289, 321. LC-MS: *m/z* = 276 [M + H]^+^. Anal. Calcd. for C_11_H_9_N_5_O_2_S: C, 47.99; H, 3.30; N, 25.44; S, 11.65. Found: C, 48.01; H, 3.34; N, 25.43; S, 11.61.

#### Ethyl (tetrazolo[1,5-c]quinazolin-5-ylthio)acetate (**7.2**)

Light-brown solid in 86.41% yield, mp 136–138°C. ^1^H NMR: δ (ppm) 8.55 (d, 1H, H-10), 7.95 (m, 2H, H-7,9), 7.82 (t, *J* = 7.32 Hz, 1H, H-8), 4.26 (s, 2H, SCH_2_), 4.20 (t, 2H, OCH_2_), 1.32 (qd, 2H, CH_3_). IR (cm^−1^): 3013, 2983, 2938, 2849, 2313, 1731, 1617, 1588, 1490, 1472, 1450, 1384, 1365, 1325, 1300, 1276, 1248, 1194, 1146, 1105, 1037, 1026, 977, 967, 901, 862, 845, 773, 713, 689, 646, 631, 604. UV (nm): 222, 248, 289, 321. LC-MS: *m/z* = 290 [M + H]^+^. Anal. Calcd. for C_12_H_11_N_5_O_2_S: C, 49.82; H, 3.83; N, 24.21; S, 11.08. Found: C, 49.84; H, 3.82; N, 24.25; S, 11.12.

#### Butyl (tetrazolo[1,5-c]quinazolin-5-ylthio)acetate (**7.3**)

White solid in yield, mp 56–57°C. ^1^H NMR (400 MHz): δ (ppm) 8.52 (d, *J* = 7.76 Hz, 1H, H-10), 8.01 (d, *J* = 7.86 Hz, 1H, H-7), 7.92 (t, *J* = 8.11 Hz, 1H, H-9), 7.83 (t, *J* = 7.46 Hz, 1H, H-8), 4.42 (s, 2H, SCH_2_), 4.16 (t, *J* = 6.33 Hz, 2H, OCH_2_), 1.65–1.53 (m, 2H, OCH_2_*CH_2_*), 1.30 (qd, *J* = 14.66, 7.22 Hz, 2H, *CH_2_*CH_3_), 0.82 (t, *J* = 7.29 Hz, 3H, CH_3_). UV (nm): 222, 248, 289, 321. LC-MS: *m/z* = 318 [M + H]^+^. Anal. Calcd. for C_14_H_15_N_5_O_2_S: C, 52.98; H, 4.76; N, 22.07; S, 10.10. Found: C, 52.95; H, 4.73; N, 22.06; S, 10.09.

#### Ethyl 2-(tetrazolo[1,5-c]quinazolin-5-ylthio)butanoate (**7.4**)

Light-brown solid in 43.32% yield, mp 78–79°C. ^1^H NMR (400 MHz): δ (ppm) 8.53 (d, *J* = 7.76 Hz, 1H, H-10), 8.14-7.92 (m, 2H, H-7,9), 7.84 (t, *J* = 7.23 Hz, 1H, H-8), 4.80 (t, *J* = 6.61 Hz, 1H, SCH), 4.23 (q, *J* = 6.92 Hz, 2H, O*CH_2_*CH_3_), 2.27-2.01 (m, 2H, CH*CH_2_*CH_3_), 1.24 (t, *J* = 6.95 Hz, 3H, OCH_2_C*H*_3_), 1.10 (t, *J* = 7.23 Hz, 3H, CHCH_2_*CH_3_*). IR (cm^−1^): 2977, 2933, 2878, 2849, 1734, 1617, 1589, 1562, 1491, 1472, 1450, 1382, 1369, 1341, 1325, 1274, 1207, 1153, 1102, 1035, 1024, 979, 968,892, 880, 871, 809, 769, 713, 687, 674,644, 604. UV (nm): 222, 248, 291, 321.LC-MS: *m/z* = 318 [M + H]^+^. Anal. Calcd. for C_14_H_15_N_5_O_2_S: C, 52.98; H, 4.76; N, 22.07; S, 10.10. Found: C, 52.95; H, 4.79; N, 22.04; S, 10.12.

#### Methyl (4-chlorophenyl)(tetrazolo[1,5-c]quinazolin-5-ylthio)acetate (**7.5**)

White solid in 85.50% yield, mp 136–138°C. ^1^H NMR (400 MHz): δ (ppm) 8.54 (d, *J* = 7,84 Hz, 1H, H-10), 8.03 (m, H-7,9), 7.85 (t, *J* = 7.07 Hz, 1H, H-8), 7.39 (q, *J* = 8.38 Hz, 4H, Ph-2,3,5,6), 5.10 (t, *J* = 7.19 Hz, 1H, SCH), 3.72 (s, 3H, OCH_3_), 3.55-3.28 (m, 2H, CH_2_Ph). IR (cm^−1^): 3099, 3072, 3046, 3000, 2981, 2952, 2846, 1735, 1617, 1589, 1558, 1530, 1491, 1475, 1447, 1433, 1410, 1384,1344, 1326, 1300, 1278, 1240, 1195, 1172, 1158, 1094, 1039, 1015, 996, 972, 918, 881, 864, 850, 836, 811, 786, 771, 713, 686, 667, 645, 631. UV (nm): 223, 249, 291, 321. LC-MS: *m/z* = 400 [M + H]^+^. Anal. Calcd. for C_18_H_14_ClN_5_O_2_S: C, 54.07; H, 3.53; N, 17.51; S, 8.02. Found: C, 54.04; H, 3.56; N, 17.53; S, 8.00.

#### Methyl (2,4-dichlorophenyl) (tetrazolo[1,5-c]quinazolin-5-ylthio)acetate (**7.6**)

Brown solid in 92.21% yield, mp 164–166°C. ^1^H NMR (500 MHz): δ (ppm) 8.53 (d, *J* = 7.74 Hz, 1H, H-10), 8.04 (d, *J* = 7.78 Hz 1H, H-7), 7.96 (t, *J* = 8.15 Hz, 1H, H-9), 7.85 (t, *J* = 7.31 Hz, 1H, H-8), 7.62-7.48 (m, 2H, Ph-5,6), 7.33 (d, *J* = 8.15 Hz, 1H, Ph-3), 5.17 (t, *J* = 7.42 Hz, 1H, SCH), 3.74 (s, 3H, OCH_3_), 3.66 (d, 7.86 Hz, 2H, SCHC*H_2_*). IR (cm^−1^): 3567, 3336, 3085, 2973, 2923, 2894, 1732, 1616, 1589, 1557, 1496, 1470, 1451, 1436, 1380, 1340, 1317, 1288, 1276, 1234, 1194, 1152, 1101, 1048, 1033, 986, 975, 963, 880, 859, 846, 819, 768,736, 713, 703, 695, 668, 629. UV (nm): 223, 249, 291, 321. LC-MS: *m/z* = 436 [M + H]^+^. Anal. Calcd. for C_18_H_11_Cl_2_N_5_O_3_S: C, 48.23; H, 2.47; N, 15.62; S, 7.15. Found: C, 48.21; H, 2.45; N, 15.65; S, 7.12.

#### Ethyl 2-(tetrazolo[1,5-c]quinazolin-5-ylthio)-3-(m-tolyl)propionate (**7.7**)

Light-brown solid in 66.72% yield, mp 136–138°C. ^1^H NMR (400 MHz): δ (ppm) 8.53 (d, *J* = 7.77 Hz, 1H, H-10), 8.01 (m, 2H, H-7,9), 7.84 (t, *J* = 7.12 Hz, 1H, H-8), 7.18 (t, *J* = 7.94 Hz, 3H, Ph-4,5,6), 7.06 (m, 2H, Ph-2), 5,03 (t, *J* = 7.12 Hz, 1H, SCH), 4.15 (qd, *J* = 8.85 Hz, 2H, OCH_2_), 3.45-3.28 (m, 2H, CH_2_Ph), 2.27 (s, 3H, PhCH_3_), 1.15 (t, *J* = 6.71 Hz, 3H, CH_2_*CH_3_*). IR (cm^−1^): 3346, 2974, 2918, 2849, 1722, 1619, 1589, 1489, 1472, 1448, 1370, 1326, 1310, 1296, 1275, 1251, 1224, 1173, 1149, 1092, 1034, 978, 966, 903, 878,859, 770, 739, 712, 699, 674, 643, 606. UV (nm): 217, 248, 291, 321. LC-MS: *m/z* = 394 [M + H]^+^. Anal. Calcd. for C_20_H_19_N_5_O_2_S: C, 61.05; H, 4.87; N, 17.80; S, 8.15. Found: C, 61.02; H, 4.85; N, 17.83; S, 8.14.

#### Methyl 4-[(tetrazolo[1,5-c]quinazolin-5-ylthio)methyl]benzoate (**7.8**)

Green-yellowish solid in 71.75% yield, mp 138–140°C. ^1^H NMR (500 MHz): δ (ppm) 8.51 (d, *J* = 7.87 Hz, 1H, H-10), 8.12 (d, *J* = 8.27 Hz, 1H, H-7), 7.92 (d, *J* = 8.39 Hz, 2H, H-7,9), 7.83 (t, *J* = 7.67 Hz, 1H, H-8), 7.79 (d, J = 8.17 Hz, 2H, Ph-3,5), 7.81 (d, *J* = 7.92 Hz, 2H), 4.85 (s, 2H, SCH_2_), 3.84 (s, 3H, OCH_3_). IR (cm^−1^): 3064, 2992, 2944, 2919, 2842, 1943, 1712, 1615, 1586, 1557, 1521, 1488, 1474, 1449, 1433, 1414, 1383, 1324, 1307, 1275, 1246, 1192, 1177, 1161, 1102, 1036, 1016, 982, 966, 885, 875, 852, 837, 807, 795, 769, 731, 731, 711, 642, 618. UV (nm): 221, 245, 289, 323. LC-MS: *m/z* = 352 [M + H]^+^. Anal. Calcd. for C_17_H_13_N_5_O_2_S: C, 58.11; H, 3.73; N, 19.93; S, 9.12. Found: C, 58.15; H, 3.70; N, 19.96; S, 9.10.

### Biological assays

#### Bioluminescence inhibition test

The marine luminescent bacteria *Photobacterium leiognathi* strain Sh1, isolated from the Azov Sea Shrimp, were used for the bioluminescence analysis. Bacteria were cultivated on a nutrient environment containing (g/L): pepton – 5, yeast extract – 1.5, meat extract – 1.5, sodium chloride – 30, pH 7.4. In the acute action test (inhibiting luminescence of bacteria), bacteria were diluted with the 3% sodium chloride solution up to the concentration 10^5^ cell/mL. The 5 – 50 μg/mL of the studied substances suspended in DMSO were mixed with 1 mL of the diluted bacterial suspension. Vials were incubating for 10 minutes at 25 °C, then the intensity of bioluminescence was measured in % relatively to the control tests which were prepared without the studied compounds. In the chronic action test (inhibiting growth and luminescence of bacteria), growth environment was added to the eventual breeding 1:50 and was incubated for 16–18 hours at 30 °C, whereupon the intensity of bioluminescence was measured as well as in the previous method. Tetracycline was used as a reference. The bacterial luminescence was measured with the Bioluminometer BLM-8801 («Science», Krasnoyarsk, Russia).

#### Antimicrobial and antifungal test

All the newly synthesized compounds were evaluated for their *in vitro* antibacterial activity in the Zaporozhye Regional Hospital Bacterial Laboratory against Gram positive bacteria (*Staphylococcus aureus* ATCC 25923, *Enterobacter aerogenes*, *E. faecalis* ATCC 29212), Gram negative bacteria (*Pseudomonas aeruginosa* ATCC 9027, *Escherichia coli* ATCC 25922, *Klebsiella pneumoniae* 68). They were also evaluated for their *in vitro* antifungal potential against *Candida albicans* ATCC 885653. The amount of microbial cells was 1.5 * 10^8^ c.f.u./mL. The incubation period of bacteria was 24 h at 35 °C, yeast – 48–72 h at 28–30°C. The agar-diffusion method was used for the determination of the preliminary antibacterial and antifungal activity. Standard sterilized filter paper discs (6 mm diameter) impregnated with a solution of the test compound in DMSO (100 μg/disk) were placed on an agar (Müller–Hinton Broth (Oxoid)) plate seeded with the appropriate test organism in triplicates. DMSO alone was used as the control at the same above-mentioned concentration. Ampicillin, Ceftazide, Amikacin, Gentamycin, Ceftriaxone, and Nystatin were used as reference drugs. The results were recorded for each tested compound as the average diameter of inhibition zones diameters (IZD) of bacterial or fungal growth around the discs in mm.

#### Antitumor activity

The primary anticancer assay was performed against the human tumor cell lines panel derived from nine neoplastic diseases, in accordance with the protocol of the Drug Evaluation Branch, National Cancer Institute, Bethesda [28–30]. The human tumor cell lines of the cancer screening panel were grown in RPMI 1640 medium containing 5% fetal bovine serum and 2 mM L-glutamine. For a typical screening experiment, cells were inoculated in 96 well microtiter plates in 100 mL assay volume, at plating densities ranging from 5000 to 40000 cell/well. After cell inoculation, the microtiter plates were incubated at 37°C under an atmosphere of 5:95 CO_2_:air (v/v) at 100% relative humidity, for 24 h prior to the addition of drugs under assessment. Following drug addition (1 μM), the plates were incubated for an additional 48 h, under the same conditions. Sulforhodamine B (SRB) solution (100 μL, 0–4% w/v in 1% aq. acetic acid) was added to each well and plates were incubated for 10 min at room temperature. The percentage growth was evaluated spectrophotometrically versus the controls not treated with test agents.

### Docking, scoring, and visual inspection of synthesized substances into ATP binding site of EGFR

Research was conducted by flexible molecular docking, as an approach for finding molecules with affinity for a specific biological target using the software package OpenEye, including related utilities: Fred Receptor2.2.5, Vida4.1.1, Flipper, Babel3, Omega2.4.3, and Fred2.2.5 [33]. The crystal structure of the enzyme EGFR (2ITY.pdb) with Gefitinib was obtained from the protein data bank [32].

The methodology of research consisted of the following steps:

○ generation of R-, S- and cis-, trans-isomers of ligands (the studied compounds and relevant drugs, program Flipper), which allowed the production isomer’s range of studied compounds;
○ generation of 3D-structure of the obtained isomeric forms - molecular modelling (Hyper Chem 7.5) using the method of molecular mechanics (MM +) and semiempirical quantum mechanical method with Polak-Ribiere algorithm (PM3);
○ generation of conformations of ligands (Omega2.4.3). The number of conformations obtained wasn’t significant due to the further selection by program Fred2.2.5 most optimal conformer;
○ carrying out molecular docking (Fred2.2.5).

A number of scoring functions (Shapegauss, PLP, Chemgauss2, Chemgauss3, Chemscore, OEChemscore, Screenscore, CGO, CGT, Zapbind, Consensus Score) was obtained as a result of the studies, values of which assess specific characteristics of the ligand-protein complex, indicating the possibility of their matching.

## Figures and Tables

**Sch. 1.**
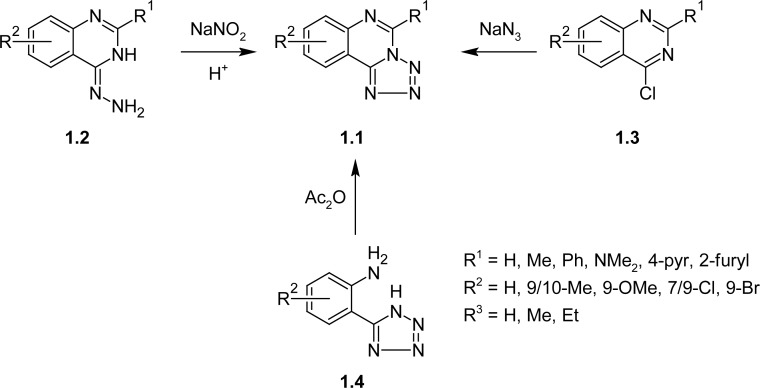
Synthesis of the tetrazolo[1,5-*c*]quinazolines

**Sch. 2.**
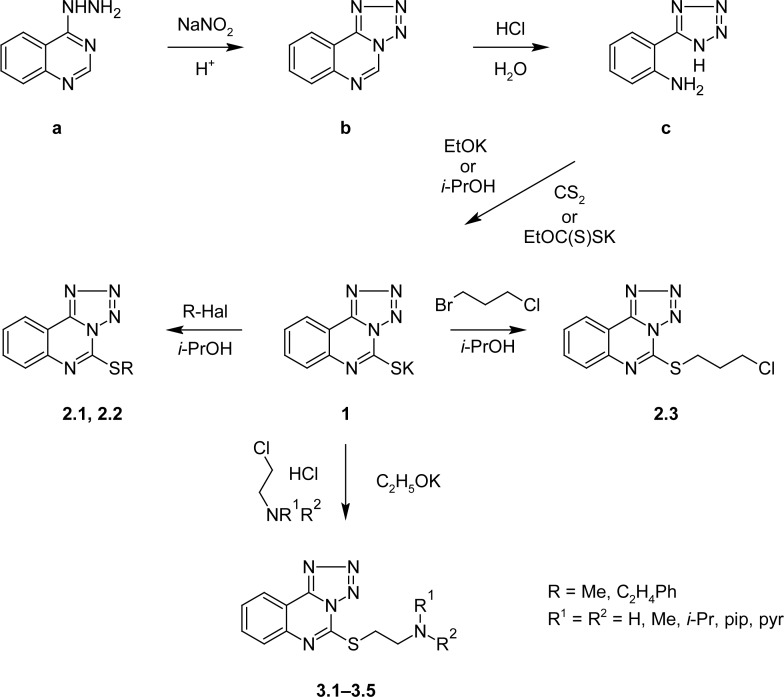
Synthesis of the tetrazolo[1,5-*c*]quinazoline-5-thione potassium salt and its S-derivatives

**Sch. 3.**
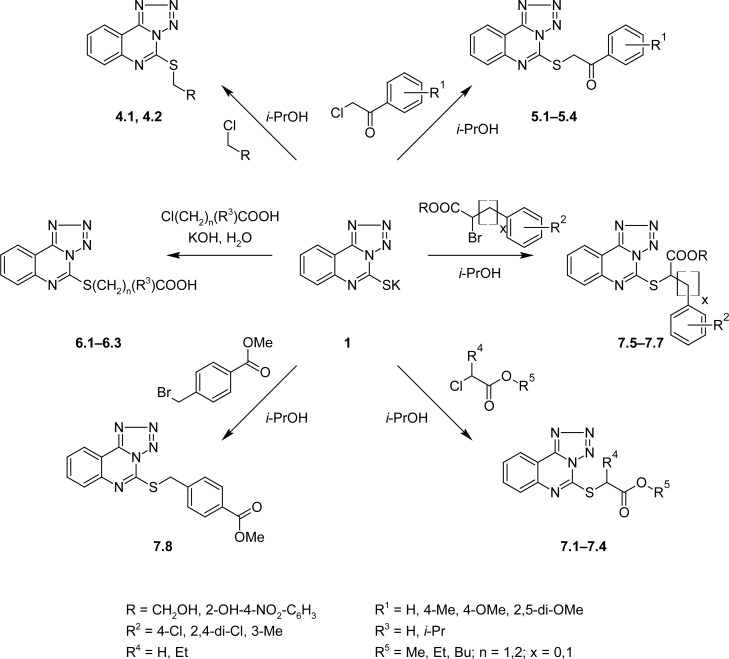
Synthesis of 2-(tetrazolo[1,5-*c*]quinazolin-5-ylthio)ethanones (**5.1–5.4**), alcohols **4.1**, **4.2**, carboxylic acids **6.1–6.3**, and carboxylic acid esters **7.1–7.8**

**Tab. 1.** Values of BL in acute action test (%)

**Compd.^a^**	**Concentration (mg/mL)**			
	**0**	**0.025**	**0.1**	**0.25**
**2.1**	100.0	63.0	30.3	27.7
**2.2**	100.0	96.0	86.0	100.0
**2.3**	100.0	37.9	22.1	18.9
**3.1**	100.0	65.9	59.0	10.4
**3.2**	100.0	41.4	15.9	3.2
**3.3**	100.0	98.9	25.6	15.3
**3.4**	100.0	46.8	6.9	5.2
**3.5**	100.0	34.8	0.0	0.0
**4.2**	100.0	9.6	3.2	3.2
**5.1**	100.0	4.6	0.0	0.0
**5.2**	100.0	3.3	0.0	0.0
**5.3**	100.0	3.5	6.9	6.9
**5.4**	100.0	1.7	0.0	0.0
**6.1**	100.0	1.7	0.0	0.0
**7.1**	100.0	47.4	7.9	3.2
**7.2**	100.0	97.1	52.0	27.7
**7.3**	100.0	90.2	26.0	8.7
**7.4**	100.0	92.5	34.7	23.1
**7.5**	100.0	106.9	46.2	34.7
**7.6**	100.0	67.9	69.5	47.4
**7.7**	100.0	77.6	64.7	43.1
**7.8**	100.0	49.4	8.5	3.4
Tetracycline	100.0	80.7	9.1	0
DMSO (control)	100.0	141.7	119.6	110.4

a substances **4.1, 6.2, 6.3** were not tested.

**Tab. 2.** Values of BL in chronic action test (%)

**Compd.^a^**	**Concentration (mg/mL)**			
	**0**	**0.025**	**0.1**	**0.25**
**2.1**	100.0	174.4	177.9	150.0
**2.2**	100.0	3.0	8.0	10.0
**2.3**	100.0	94.4	236.0	337.1
**3.1**	100.0	66.7	27.8	11.1
**3.2**	100.0	122.2	20.8	3.7
**3.3**	100.0	271.4	0.0	85.7
**3.4**	100.0	77.8	88.9	100.0
**3.5**	100.0	171.4	0.0	0.0
**4.2**	100.0	66.0	29.3	2.4
**5.1**	100.0	35.5	0.0	0.0
**5.2**	100.0	0.0	0.0	0.0
**5.3**	100.0	0.0	0.0	0.0
**5.4**	100.0	142.9	0.0	0.0
**6.1**	100.0	0.0	0.0	0.0
**7.1**	100.0	303.4	337.1	337.1
**7.3**	100.0	55.6	244.4	22.2
**7.4**	100.0	100.0	25.0	20.0
**7.5**	100.0	62.0	24.0	22.0
**7.6**	100.0	215.7	209.0	327.0
**7.7**	100.0	100.0	100.0	100.0
**7.8**	100.0	207.1	214.3	521.4
Tetracycline	100.0	0	0	0
DMSO (control)	100.0	74.5	127.7	127.7

a substances **4.1, 6.2, 6.3, 7.2** were not tested.

**Tab. 3.** Antimicrobial activity of compounds (100 μg)

**Compd.**	**Conc., μg**	**Microorganism / Inhibition zone diameter (mm)**						
		***EC*^a^**	***SA***	***EA***	***EF***	***PA***	***KP***	***CA***
**2.3**	100	6	6	6	6	6	6	22
**3.1**	100	15	14	6	15	6	6	15
**3.2**	100	15	12	6	16	6	6	16
**3.3**	100	6	11	6	18	6	6	10
**4.2**	100	6	7	6	6	6	6	6
**5.4**	100	21	30	6	6	16	11	22
Ampicillin	10	26	22	16	17	–	–	–
Ceftazide	30	29	21	–	–	25	–	–
Amikacin	30	21	22	–	–	27	–	–
Gentamycin	10	19	22	7	9	28	–	–
Ceftriaxone	30	33	33	–	–	25	25	–
Nystatin	100	–	–	–	–	–	–	21

a *Escherichia coli (EC)*, *Staphylococcus aureus (SA)*, *Enterobacter aerogenes (EA)*, *Enterococcus faecalis (EF)*, *Pseudomonas aeruginosa (PA)*, *Klebsiella pneumoniae (KP)*, *Candida albicans (CA)*; «6 mm» – disk diameter; «–» not done. Synthesized substances, except **2.3**, **3.1**–**3.3**, **4.2**, **5.4,** in researched concentrations exhibited no antimicrobial and antifungal activities.

**Tab. 4.** Percentage of *in vitro* tumor cell lines growth at 10 μM

**Subpanel tumor cell lines**	**Compound / Growth Percent**									
	**2.1**	**2.2**	**6.1**	**6.2**	**6.3**	**5.1**	**5.2**	**5.3**	**5.4**	**7.2**
**Leukemia**										

CCRF-CEM	97.44	92.02	107.51	**−44.08**	**−55.61**	92.90	**−49.78**	91.76	88.15	**−32.45**
HL-60(TB)	101.99	79.15	103.36	106.51	**41.25**	104.16	**36.80**	90.36	97.94	nt^a^
K-562	101.22	97.87	86.32	98.17	105.93	86.61	96.93	97.00	99.63	nt
MOLT-4	93.11	81.16	96.01	91.19	**46.20**	86.39	nt	84.62	99.88	nt
RPMI-8226	104.88	101.73	101.50	84.07	99.05	101.79	99.28	96.57	102.79	87.95
SR	88.13	88.31	97.02	96.95	96.08	86.13	91.09	92.71	112.94	nt

**Non small cell lung**										

A549/ATCC	103.92	91.35	98.41	92.60	98.28	102.51	99.26	96.11	132.78	91.08
EKVX	133.29	89.82	101.82	89.92	99.94	101.63	101.68	100.08	107.14	98.65
HOP-62	88.90	115.57	96.76	92.62	96.49	85.82	102.54	108.35	79.16	105.21
HOP-92	103.41	81.81	98.54	96.39	112.41	110.33	111.47	105.30	75.10	98.85
NCI-H226	94.93	96.84	92.24	107.47	108.67	95.48	108.46	100.00	92.90	109.38
NCI-H23	101.76	98.09	99.56	95.88	104.61	98.53	106.86	108.47	96.48	97.04
NCI-H322M	88.30	90.71	90.86	112.22	127.43	95.83	137.89	89.45	89.14	125.60
NCI-H460	121.91	103.19	109.41	105.88	111.77	90.95	99.52	115.45	**69.70**	103.91
NCI-H522	100.17	86.12	102.55	119.63	149.77	90.38	102.52	95.22	119.32	103.35

**Colon cancer**										

COLO 205	126.87	135.56	114.99	111.49	117.16	113.86	109.22	126.66	137.77	119.03
HCC-2998	122.55	103.09	104.34	113.48	102.59	102.68	101.20	107.93	112.35	99.57
HCT-116	99.74	99.35	106.33	103.97	110.06	100.22	112.04	110.24	91.53	110.69
HCT-15	117.55	98.58	121.37	104.97	111.17	105.80	104.06	108.15	108.94	103.94
HT29	nt	nt	nt	nt	nt	99.98	nt	nt	106.25	nt
KM12	109.63	96.00	107.22	105.13	110.45	101.53	98.86	112.12	81.85	106.24
SW-620	119.18	107.02	110.29	99.04	102.97	108.53	105.69	114.43	116.56	101.58

**CNS cancer**										

SF-268	116.78	107.46	116.08	116.33	109.69	116.44	108.56	108.41	90.65	107.38
SF-295	153.32	96.99	103.88	96.58	135.09	94.86	104.68	nt	115.90	102.37
SF-539	104.74	98.74	110.65	95.53	89.37	92.26	104.58	103.26	82.18	94.52
SNB-19	99.20	94.40	112.47	102.67	107.54	96.55	109.90	105.13	75.54	102.05
SNB-75	**63.81**	87.92	83.62	86.15	93.93	70.77	88.80	84.89	**55.07**	92.20
U251	103.03	97.20	95.31	97.96	99.79	84.99	93.10	103.85	80.57	97.98

**Melanoma**										

LOX IMVI	101.34	96.42	100.69	92.90	116.03	94.45	96.37	112.71	89.85	93.29
MALME-3M	95.64	109.38	90.36	114.34	119.66	76.35	142.48	95.17	78.16	105.87
M14	nt	nt	nt	99.46	105.65	nt	108.01	nt	nt	107.70
MDA-MB-435	114.98	104.24	120.44	97.27	103.16	96.43	93.17	110.45	89.28	102.60
SK-MEL-2	100.82	109.83	107.03	96.85	148.79	97.34	112.39	106.13	126.88	122.15
SK-MEL-28	110.40	103.09	109.54	102.83	112.55	99.40	102.99	110.38	92.93	108.22
SK-MEL-5	93.48	92.89	99.35	104.74	109.69	98.84	109.31	99.50	90.07	106.19
UACC-257	122.47	109.50	101.37	99.16	107.83	95.53	107.98	105.99	147.08	99.58
UACC-62	90.48	80.69	101.67	99.08	98.44	93.55	93.60	104.14	95.02	97.27

**Ovarian cancer**										

IGROV1	91.01	85.40	96.15	111.14	126.33	91.28	115.60	88.62	81.47	111.11
OVCAR-3	112.33	106.17	115.78	120.17	119.02	118.24	122.02	128.65	110.65	120.07
OVCAR-4	104.59	87.46	102.78	105.19	112.42	91.34	100.96	101.12	79.56	105.94
OVCAR-5	109.29	114.14	119.67	97.83	101.33	109.81	114.69	111.83	110.65	108.78
OVCAR-8	102.07	101.20	105.58	103.41	102.64	98.84	108.10	99.96	124.93	98.54
NCI/ADR-RES	113.65	102.41	106.02	101.68	107.97	98.70	106.62	106.04	84.71	98.07
SK-OV-3	111.47	109.97	111.28	nt	110.22	89.90	104.01	115.04	76.63	nt

**Renal cancer**										

786-0	101.89	98.61	103.47	104.96	112.93	103.85	112.33	104.52	97.77	109.51
A498	108.08	94.35	102.37	110.63	110.41	105.50	100.02	116.57	95.62	100.34
ACHN	106.37	106.39	106.11	100.00	102.31	103.42	114.11	112.05	104.37	105.78
CAKI-1	102.57	85.20	92.27	87.91	96.79	101.63	91.92	98.72	102.19	95.23
RXF 393	104.74	101.27	106.23	116.17	117.62	96.11	123.68	110.51	87.85	118.03
SN12C	91.02	93.84	102.06	92.16	105.11	89.04	99.14	105.19	91.11	98.15
TK-10	126.69	118.06	109.98	114.15	143.09	126.60	111.92	115.61	143.85	109.41
UO-31	**61.31**	82.27	70.02	75.96	97.25	**63.82**	99.42	78.32	81.56	85.29

**Prostate cancer**										

PC-3	98.24	84.16	102.80	103.40	110.71	93.79	101.84	102.91	87.85	99.73
DU-145	116.79	113.12	113.39	113.87	120.10	112.32	116.29	121.38	101.11	110.60

**Breast cancer**										

MCF7	90.60	86.74	95.61	106.88	112.32	86.66	98.18	85.87	73.73	100.14
MDA-MB-231/ATCC	nt	nt	nt	91.60	103.02	nt	106.33	nt	nt	91.02
HS 578T	135.25	118.35	112.55	99.29	100.43	115.26	94.20	128.13	88.33	101.94
BT-549	81.64	91.94	107.80	102.08	115.97	96.76	113.47	115.36	96.25	111.83
T-47D	111.24	98.90	113.38	102.44	126.40	101.67	106.17	117.56	98.33	105.02
MDA-MB-468	92.87	73.60	97.72	109.35	110.48	90.51	115.79	98.08	86.67	119.10

a nt…not tested.

**Tab. 5.** Molecular docking scoring functions of compounds with anticancer activity results (**2.1, 2.2, 5.1–5.4, 6.1–6.3, 7.1**) versus Gefitinib, Lapatinib data

**Compd.**	**Consensus Score**	**Shapegauss**	**PLP**	**Chemgauss2**	**Chemgauss3**	**Chemscore**
**Gefitinib**	149.00	−507.26	−56.55	−59.78	−69.13	−20.41
**Lapatinib**	175.00	−599.98	−61.50	−64.46	− 71.10	−16.22
**6.2**	651.00	−377.77	−43.14	−41.16	−60.63	−17.81
**5.1**	656.00	−429.12	−42.17	−41.20	−61.46	−15.20
**6.3**	775.00	−379.88	−33.19	−37.20	−54.66	−12.79
**5.2**	780.00	−377.46	−32.03	−36.82	−44.99	−12.22
**5.3**	781.00	−381.74	−31.89	−36.96	− 41.17	−11.92
**7.1**	804.00	−353.99	−28.91	−36.26	− 54.69	− 9.99
**5.4**	814.00	−408.72	− 36.84	− 41.84	− 47.94	− 13.46
**2.2**	843.00	−370.02	− 37.31	− 35.79	− 43.00	− 14.79
**2.1**	900.00	−299.81	− 28.92	− 32.30	− 51.36	− 10.98
**6.1**	903.00	−327.41	− 27.35	− 32.33	− 43.18	− 11.12

**Compd.**	**OEChemscore**	**Screenscore**	**CGO**	**CGT**	**Zapbind**	
	
**Gefitinib**	−35.31	−126.06	−367.45	−0.61	−25.60	
**Lapatinib**	−42.82	−131.70	−329.62	−0.43	−29.62	
**6.2**	−27.11	−90.14	−272.08	−0.60	−17.84	
**5.1**	−26.30	−103.58	−282.51	−0.52	−21.15	
**6.3**	−22.82	−82.92	−298.63	−0.57	−22.85	
**5.2**	−21.23	−89.15	−331.98	−0.54	−23.66	
**5.3**	−20.44	−88.76	−346.31	−0.56	−23.73	
**7.1**	−19.25	−72.56	−291.77	−0.59	−23.48	
**5.4**	−24.48	−87.05	−291.86	−0.42	−21.50	
**2.2**	−23.13	−87.50	−254.17	−0.45	−24.97	
**2.1**	−18.41	−69.03	−222.46	−0.47	−16.88	
**6.1**	−19.81	−74.55	−259.17	−0.52	−16.88	
